# A Highly Sensitive Strain Sensor with Self-Assembled MXene/Multi-Walled Carbon Nanotube Sliding Networks for Gesture Recognition

**DOI:** 10.3390/mi15111301

**Published:** 2024-10-25

**Authors:** Fei Wang, Hongchen Yu, Xingyu Ma, Xue Lv, Yijian Liu, Hanning Wang, Zhicheng Wang, Da Chen

**Affiliations:** Laboratory for Intelligent Flexible Electronics, College of Electronic and Information Engineering, Shandong University of Science and Technology, Qingdao 266590, China; yuhc0207@163.com (H.Y.); mxy18253103525@163.com (X.M.); lxsdkd@163.com (X.L.); liuyijian@sdust.edu.cn (Y.L.); wangcc0906@163.com (H.W.); wangzhicheng320@163.com (Z.W.)

**Keywords:** MXene/MWCNT strain sensor, self-assembly, human body detection, deep learning

## Abstract

Flexible electronics is pursuing a new generation of electronic skin and human–computer interaction. However, effectively detecting large dynamic ranges and highly sensitive human movements remains a challenge. In this study, flexible strain sensors with a self-assembled PDMS/MXene/MWCNT structure are fabricated, in which MXene particles are wrapped and bridged by dense MWCNTs, forming complex sliding conductive networks. Therefore, the strain sensor possesses an impressive sensitivity (gauge factor = 646) and 40% response range. Moreover, a fast response time of 280 ms and detection limit of 0.05% are achieved. The high performance enables good prospects in human detection, like human movement and pulse signals for healthcare. It is also applied to wearable smart data gloves, in which the CNN algorithm is utilized to identify 15 gestures, and the final recognition rate is up to 95%. This comprehensive performance strain sensor is designed for a wide array of human body detection applications and wearable intelligent systems.

## 1. Introduction

Flexible electronics are driving the development of a new generation of electronic skin [1,2,3,4] and human–machine exchange [5,6,7]. Therefore, there is an urgent need to attain high sensitivity, a wide sensing range, and exceptional durability in contemporary devices [8,9,10,11]. Particularly, the detection of small strain signals and the highly sensitive monitoring of human motion continue to pose significant challenges for wearable sensors. Although flexible strain sensors have been continuously innovating over the past decades [12,13,14], there are still limitations restricting their performance. Considerable efforts have been made to achieving an ideal flexible strain sensor, in which novel materials with excellent conductivity and compatibility are utilized, such as metal nanoparticles [15,16,17], carbon nanotubes [18,19,20,21,22], carbon nanofibers [23,24,25], reduced graphene oxide [26,27,28], and MXene [29,30,31]. Thereinto, MXene materials are potential candidates with a layered 2D structure, which could achieve significant resistance variations for high-level sensitivity in MXene-based sensors [32,33], but their narrow response range remains a vital challenge in flexible strain sensors.

Considering the requirements mentioned above, complementary materials and an optimized sensing structure should be further employed to overcome restrictions. Due to their distinctive spiral architecture and elevated aspect ratio, multi-walled carbon nanotubes (MWCNTs) play a pivotal role in augmenting the range of strain sensing [34,35]. Thus, sensors based on composite materials of MXene and MWCNTs with a preferable sensitivity and a wide sensing range [36,37] are continuously considered. Most research contributes to modifying the properties of MXene and MWCNT materials to improve sensors’ performance [38,39], but the mere combination of the materials is inadequate for practical applications [40]. On the other hand, as for sensing structure, the elastic-sliding conductive network is regarded as a significant method for the performance enhancement of flexible strain sensors [41,42], whereas MXene/MWCNT flexible strain sensors based on the elastic-sliding conductive network have been little studied. Furthermore, sensors featuring self-assembled structures can enhance mechanical strength and durability by leveraging the synergistic interactions among their layers, thus maintaining sensing performance under extreme conditions [43]. Comprehensively, the combination of MXene/MWCNTs with a self-assembled elastic-sliding conductive network is expected to be favorable to performance improvement, but few detailed analyses have revealed their synergetic mechanism. Thus, it is important to systematically analyze the mechanism, and it is equally important to find their potential applications, which is beneficial to flexible strain sensors.

In this work, we performed systematic research on flexible strain sensors with self-assembled MXene/MWCNTs and a flexible polydimethylsiloxane (PDMS) substrate, in which MXene particles are wrapped and bridged by dense MWCNTs forming complex sliding conductive networks, resulting in a good relative resistance change in both small and large strain tensions, and it is crucial to emphasize that this material exhibits a high sensitivity, characterized by a GF of 646 within the strain range of 20% to 40%. Furthermore, it demonstrates a response range extending up to 40%, with a response time of 280 ms and a detection limit as low as 0.05%. With high performance, the flexible strain sensor enables effective human body detection and gesture recognition, which is significant for future development and application.

## 2. Experimental

### 2.1. Material

The MWCNTs (diameter = 3–15 µm, average length = 15–30 µm, and >95% purity) were provided by Hongdakang Evolution Technology Co., Ltd., Shenzhen, China. The MXene was provided by Guangdong Foshan Xinxi Technology Co., Ltd., Guangdong, China. The PDMS and its curing agent (Sylgard 184 Silicone Elastomer Kit) were purchased from Dow Corning Co., Ltd., Michigan, MI, USA. The diallyldimethylammonium chloride (PDDA, PDLS 35, and polycation) was purchased from Wuxi Tianxin Chemical Co., Ltd., Jiangsu, China. The 4-styrenesulfonate (PSS) (average Mw ≈ 70,000, polyanion) was provided by Shanghai Yuanye Biotechnology Co., Ltd., Shanghai, China. The sodium dodecyl sulfate (SDS) was purchased from Kena Carbon New Materials Co., LTd., Xiamen, China.

### 2.2. Instruments

The deionized water machine used was the UPTA-20 from Lichen Instrument Technology Co., Ltd. (Zhejiang, China), and the ultrasonic cleaner uses was the JP-080S from Jiemeng Technology Co., Ltd. (Chongqing, China). The 12A spin-coater used was the KW-4A of the crown electronic equipment factory. The plasma cleaning machine used was the PT-2S from Sanhe Boda Electromechanical Technology Co., Ltd. (Shenzhen, China). The desktop electric constant temperature blast drying oven used was the DHG-9123A from Shanghai Jinghong Equipment Co., Ltd. (Shanghai, China).

### 2.3. Preparation of MXene and MWCNT Suspension

**Figure 1:**
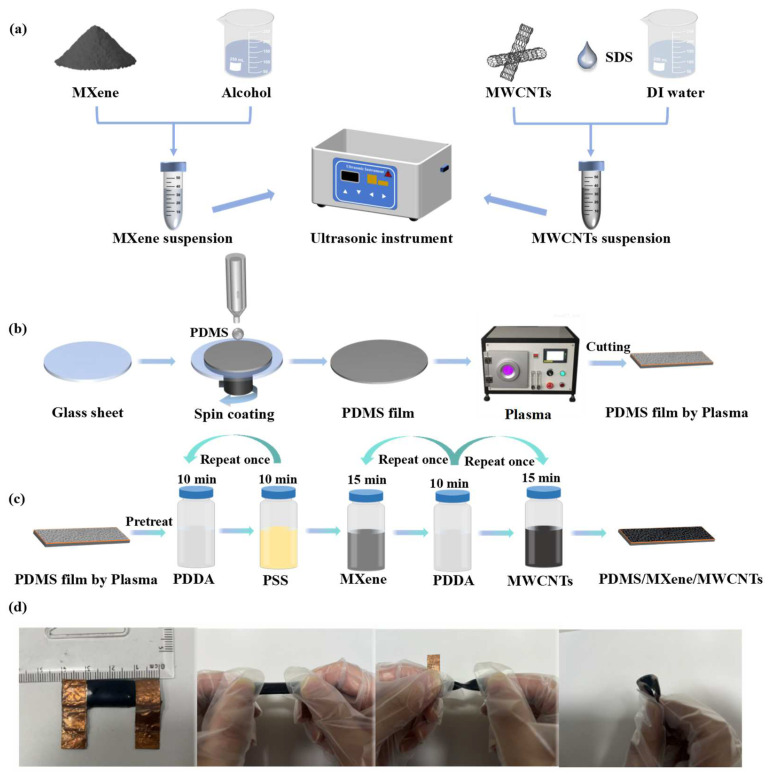
The preparation method of PDMS/MXene/MWCNT strain sensor. (**a**) The preparation of MXene and MWCNT solutions. (**b**) The PDMS films prepared by plasma treatment. (**c**) The procedure of the self-assembling method to prepare the conducting layers. (**d**) An actual image of PDMS/MXene/MWCNT strain sensor and the images of the sensor stretched, twisted, and folded.

Firstly, 2 g of MXene powder and 4 mL of absolute ethanol were continuously ground in the grinding body for 15 min, and then the resulting abrasive was thoroughly mixed with 100 mL of absolute ethanol and treated by sonication for 2 h to ensure that the material was fully dispersed to obtain the MXene dispersion. At the same time, 2 g of MWCNT powder and 1.5 g of dispersion (SDS) were added to 100 mL of deionized water and treated by sonication for 2 h to obtain the MWCNT dispersion. The weight percentages (wt%) of the two solutions were 2.3% and 1.9%, respectively. The preparation process is shown in Figure 1a.

### 2.4. Preparation of PDMS Film by Plasma

The PDMS and its curing agent (Sylgard 184 Silicone Elastomer Kit) were combined in a mass ratio of 10:1. The resulting mixture was then spin coated onto a glass substrate at a speed of 50 rpm for 10 s, followed by curing at 70 °C for 2 h to yield a new PDMS film. Subsequently, the obtained PDMS film underwent oxygen plasma treatment for 30 s. Oxygen plasma-treated PDMS films are more likely to adsorb ions. Figure 1b illustrates the preparation of the oxygen plasma-treated PDMS films.

### 2.5. Fabrication of PDMS/MXene/MWCNT Strain Sensor

The process of fabricating the PDMS/MXene/MWCNT strain sensor is illustrated in Figure 1c. The plasma-treated PDMS membrane was immersed in 1.5 wt% PDDA solution for 10 min to obtain enough positive charge and then immersed in 0.5 wt% PSS solution for 10 min to obtain a negative charge. Subsequently, the process was repeated two times until a PDDA/PSS structure was formed to capture more ions. The PDMS films were soaked in the MXene solution for 15 min and then placed in the PDDA solution for 10 min before being soaked in the MWCNTs and the PDDA solution in the same way. After the soaking was completed, the process of self-assembling a layer was completed. The soaking process was repeated to increase the layers of self-assembly. After the self-assembly step was completed, the PDMS film was attached to the copper foil board using silver paste as an adhesive and further fastened with polyurethane tape. With the completion of the connection, the PDMS and its curing agent, mixed in a mass ratio of 10:1, were applied to both sides of the sensing layer. The assembly was then cured at 70 °C for 2 h, effectively protecting the sensing layer from external disturbances such as friction, humidity, and material shedding. The final prepared PDMS/MXene/MWCNT strain sensor is shown in Figure 1d, and it can be seen that the sensor can be well stretched, twisted, and folded. This indicates that the sensor exhibits excellent flexibility and adhesion to skin surfaces.

### 2.6. Characterization

A scanning electron microscope (SEM) (TESCAN MIRA LMS, Brno, Czech Republic) was used to capture the top SEM view of the PDMS/MXene/MWCNT strain sensor. Raman spectra of the MWCNT, MXene, and MXene/MWCNT conductive layers were acquired utilizing a Raman spectrometer (Horiba LabRAM HR Evolution, Horiba, Japan). XRD patterns of the MWCNTs MXene, and MXene/MWCNT conductive layers were obtained using an X-ray diffractometer (Rigaku SmartLab SE, Tokyo, Japan). The resistance change in the PDMS/MXene/MWCNT strain sensor was recorded with a high-precision digital multimeter (Rigol DM3068 6.5 Digits, Suzhou, China) and a data acquisition instrument (KeySight DAQ970A, California, CA, USA). The relative resistance change (ΔR/R_0_ = (R − R_0_)/R_0_) and the gauge factor (GF = (R − R_0_)/R_0_ε) were calculated respectively, where R is the resistance of the applied strain; R_0_ is the initial resistance of the sensor; and ε is the deformation of the sensor.

## 3. Results and Discussion

**Figure 2:**
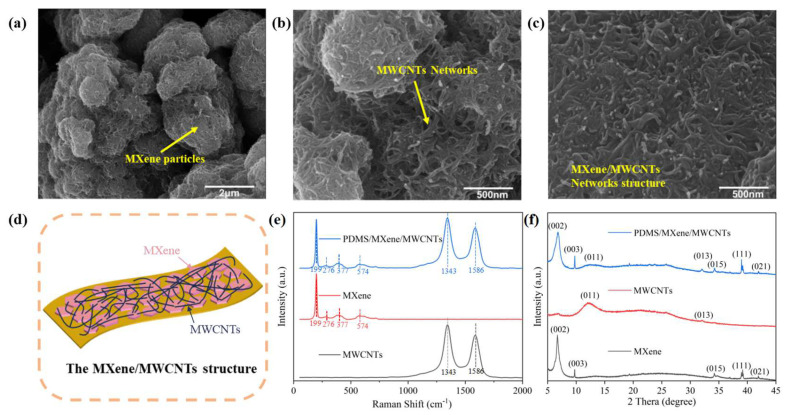
(**a**–**c**) The top-view SEM images of the PDMS/MXene/MWCNT strain sensor. (**d**) The schematic representation of the MXene/MWCNT structure. (**e**) The Raman spectra and (**f**) X-ray diffraction (XRD) results for MWCNTs, MXene, and the conductive layers composed of MXene/MWCNTs.

In order to research the self-assembled MXene/MWCNTs clearly, the top-view SEM images are shown in Figure 2a–c, in which the MXene particles and MWCNT network can be observed. Moreover, as for the composite material of MXene/MWCNT, MXene particles are wrapped and bridged by dense MWCNTs forming complex sliding conductive networks, wherein the MWCNT networks play an important role in connecting MXene particles during stretching and releasing. The sliding mechanism of this island–bridge structure is illustrated in the schematic diagram (Figure 2d) for further comprehension. The Raman spectroscopy is shown in Figure 2e with two obvious peaks (at 1336 and 1573 cm^−1^) of MWCNTs and four predominant peaks (at 199, 276, 377, and 574 cm^−1^) of the MXene, respectively. The curve of the PDMS/MXene/MWCNT film possesses all the feature peaks corresponding to the aforementioned results, wherein the uniform MXene/MWCNT composite material is well formed. The XRD is also measured as shown in Figure 2f, whose results illustrate the homogeneity of MXene/MWCNTs. These results indicate that MXene/MWCNTs with the island–bridge structure possess homogeneous conductive networks for strain sensors.

**Figure 3:**
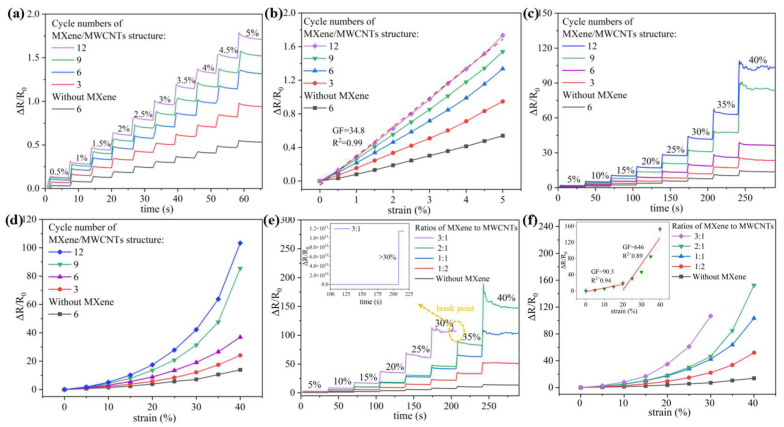
(**a**) The real-time response and (**b**) sensitivity under the gradually increasing micro-strain step of 0–5%. (**c**) The real-time response and (**d**) sensitivity of the strain sensors under a gradually increasing load, exhibiting a strain range from 0% to 40%. (**e**) The real-time response and (**f**) sensitivity of the strain sensors with varying ratios of MXene to MWCNTs. The only variable in (**a**–**d**) is the number of self-assembled layers, and (**e**–**f**) are based on sensors with 12 cycle self-assembled layers, whose variable is the ratio of materials.

To examine the optimal performance of the sensor, the real-time relative resistance change curve and the sensitivity curve of the strain sensors are illustrated in Figure 3, in which sensors with different self-assembled layers and MXene/MWCNT contents are systematically studied. The experimental method adopts the method of uniaxial vertical stretching. The performance trend of different self-assembly layers can be clearly studied in Figure 3a–d. In Figure 3a, the real-time relative resistance curves of sensors with 3–12 assembled layers of MXene/MWCNTs and a sensor with six cycles of pure MWCNTs were studied under a small strain step for different layers. It was found that the sensors with the MXene/MWCNT composite structure were observed to have a higher relative resistance change than the pure MWCNT sensor at a gradually increasing micro-strain step of 0–5%, where the resistance increased immediately and gradually stabilized with each drawing cycle. Due to the sliding effect within the conductive network, the relative changes in resistance are amplified as the assembly period increases. The average value of resistance change at each stage of strain stabilization is utilized as the representative resistance change for subsequent calculations. The corresponding sensitivity is illustrated in Figure 3b, where the GF of the sensor composed of 12 layers of self-assembled MXene/MWCNTs reaches 34.8, demonstrating a high linearity coefficient of 0.99. This indicates that the sensor is applicable to a range of micro-strain scenarios and can provide accurate output. Under large strain, from 0 to 40%, the MXene particles in the sensor layer are surrounded by dense MWCNTs, which play an important role in bridging the MWCNT network. High relative resistance changes and sensitivity are shown in Figure 3c–d, with regular curves similar to those obtained under small strain. In addition, when the strain exceeds 20%, the sensitivity increases rapidly because the MWCNTs filled between the MXene sheets decrease and even collapse, resulting in a rapid reduction in conductivity, which leads to high sensitivity at large strains [44].

At the same time, as the number of the self-assembled layers increases, the conductive filler loadings of MXene and MWCNTs on the PDMS substrate also increase, and a greater number of conductive pathways are established due to the denser material. Consequently, the initial resistance will also decrease, which indirectly proves the complicated micro texture. As the strain increases, the denser and more complex conductive networks are more likely to collapse and break, so the sensitivity increases as the number of self-assembled layers increases. Therefore, the 12-layer self-assembled strain sensor was selected for testing in the subsequent basic performance testing and application part of this experiment. Synthetically, these results indicate that the MXene/MWCNT complex sliding conductive networks provide favorable effects on strain sensitivity. For further investigating the effects of MXene content on performance, strain sensors with different ratios of MXene/MWCNTs are fabricated with 12 assembly cycle numbers in Figure 3e–f. Concentrations of 2.3 wt% MXene solution and 1.9 wt% MWCNT solution were used as the initial solution concentrations. It was found that the sensitivity increased significantly with the increase in MXene content. Interestingly, when the strain was higher than 30%, there was a breaking point in the sensor with a ratio of 3 to 1 MXene/MWCNTs in Figure 3e. This phenomenon may be due to an excess of MXene particles, which causes the separated sliding conductive network to reduce the sensing range. In addition, as demonstrated in Figure 3f, the 2:1 strain sensor displays two distinct sensitivity characteristics. Specifically, a GF of 90.3 is observed within the 0–20% strain range, while a significantly higher GF of 646 is noted in the 20–40% sensing range, which is an impressive sensitivity for a flexible strain sensor. Consequently, a strain sensor with a material ratio of 2:1 was selected for testing in the follow-up basic performance testing and application part of this experiment.

**Figure 4:**
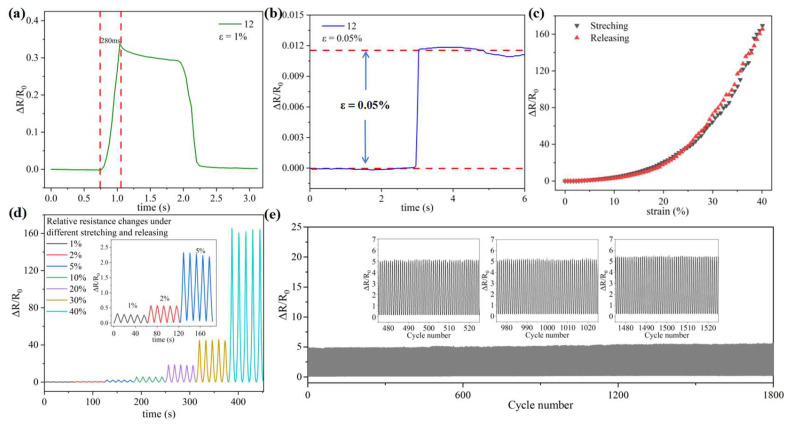
(**a**) The response time under a strain of 1%. (**b**) The relative resistance changes as a function of time under a minimal strain of 0.05%. (**c**) The real-time relative resistance response curve of the strain sensor during stretching and releasing. (**d**) The cyclic variation in relative resistance of the strain sensors subjected to different strains. (**e**) The long-term durability test with 1800 stretch and release cycles under a 10% strain.

Based on the 2:1 ratio of MXene to MWCNTs in the sensor and following 12 assembly cycles, we further evaluated the sensing performance. The results are presented in Figure 4. As illustrated in Figure 4a, the sensor exhibits a rapid response time of 280 ms under a strain of 1%, which is advantageous for real-time detection and monitoring of human physiological signals. Furthermore, as demonstrated in Figure 4b, a detection limit of 0.05% strain has been achieved, which is essential for identifying weak signals. The real-time relative resistance changes during stretching and releasing are presented in Figure 4c; the consistency between the stretch and release curves indicates that the sensor possesses high recoverability. Figure 4d shows the relative resistance at different strains varying from 1 to 40% over five cycles, where stable sensing performance is achieved under both small and large deformations. To further investigate the cyclic stability of the sensor, long-term durability tests comprising 1800 stretch and release cycles were conducted under a strain of 10%, as illustrated in Figure 4e. As the number of cycles increased, a segment of the conductive network underwent irreversible rupture. Moreover, it is worth mentioning that although there is a slight tendency to increase the resistance variation, the device still has reliability, stability, and durability. Therefore, these PDMS/MXene/MWCNT strain sensors exhibit promising sensing performance characterized by high sensitivity, rapid response time, minimal detection limits, and exceptional cyclic stability. These attributes are advantageous for practical applications. 

Table 1 lists the key parameters (including response range, sensitivity, and sensing repeatability) for several MXene or MWCNT material sensors. Compared to the above references, the PDMS/MXene/MWCNT strain transducer can have both a good response range and a good sensitivity and also has a 1800 sensing repeatability, which indicates the superiority of the sensor’s performance in our work and proves the effectiveness of the designed structure.

**Figure 5:**
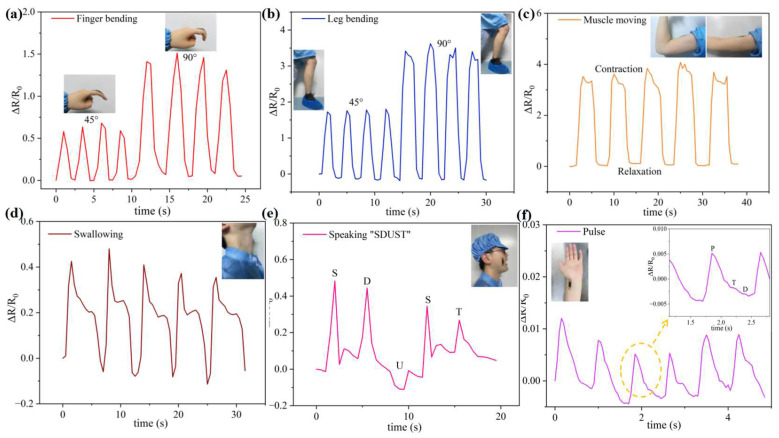
In the PDMS/MXene/MWCNT strain sensor, the relative changes in resistance were measured on (**a**) the finger, (**b**) the leg, (**c**) the muscle, and (**d**) the throat. (**e**) The sensing performance recorded during speaking “S D U S T”. (**f**) The pulse signal in the strain sensor.

Based on the results discussed above, the strain sensor is suitable for detecting both significant and subtle movements of the human body. Figure 5a–c illustrate the variations in relative resistance during finger bending, leg bending, and large muscle movements. Notably, enhanced response signals are observed with increasing motion amplitude, thereby providing strong evidence for the sensor’s detection capability. In addition, considering the ultra-low detection limit of 0.05%, subtle changes or small strains in the human body can also be detected, as shown in Figure 5d–f. For example, in Figure 5d, various electrical orders swallowed can be observed with a sensor placed on the throat. In addition, sensors are mounted on the face during different articulation processes. When the speaker articulates the letters “S D U S T” (Figure 5e), the resistance curve can be distinctly identified by the varying amplitude of motion corresponding to each letter sound. In addition, for the two instances of the “S” sound, the similar relative resistance changes regularly and steadily, indicating that the sensor can also classify the same letter. Notably, based on the small strain detection, a healthcare application could be actualized via attaching the sensors at the pulse point. The pulse detection is illustrated in Figure 5f, wherein the P, T, and D waves of pulse signals were distinguished, as in a previous work [54], proving the validity of detection in healthcare. The recorded signals offer a precise assessment of the efficacy of human motion and healthcare detection.

**Figure 6:**
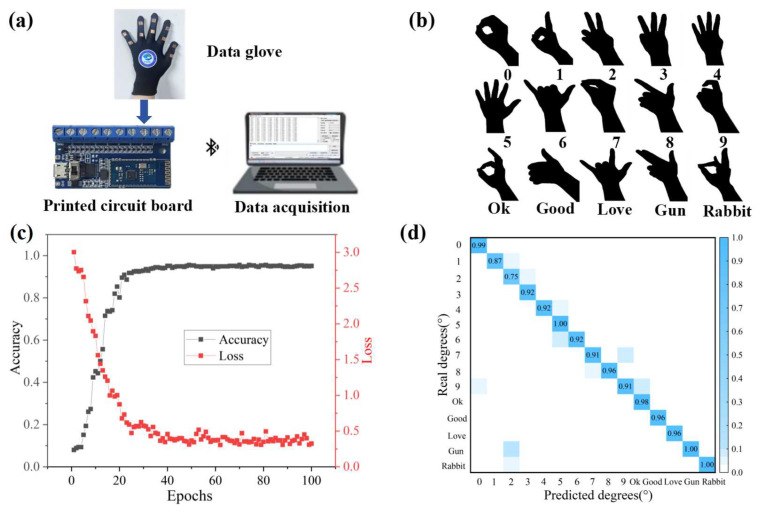
(**a**) The conceptual diagram of the designed data glove. (**b**) The actual image of the data glove displaying 15 different gestures. (**c**) The evolution process of accuracy and training loss during 100 epochs. (**d**) The confusion matrix illustrating the prediction outcomes generated by the CNN model.

Assisted by the convolutional neural network (CNN) model algorithm, the sensor could also be utilized in data gloves for the automatic intelligent classification and recognition of different hand gestures. The diagrammatic sketch is presented in Figure 6a, illustrating the sensor affixed to the glove. The EV81C octal data acquisition board is used to collect the voltage at both ends of the sensor through a STM32F030F4P6 chip and transmit the collected data to a computer in real time via Bluetooth, which helps to create a training dataset for the CNN model. The experiment performed 15 different gestures as shown in Figure 6b, such as 0 to 9, Ok, Good, Love, Gun, and Rabbit, respectively. It is noteworthy that the training loss decreases rapidly and then stabilizes to a certain value, wherein the corresponding classification accuracy after 100 training epochs was up to 95% in Figure 6c. In Figure 6d, the confusion matrix illustrating the outcomes of 15 gesture recognition tasks based on the CNN is presented. Each category demonstrates a high level of prediction accuracy, attributable to its strong generalization capability. Thus, the effective application potential of the sensor in data gloves depicts that this sensor could be utilized and coupled with machine learning for intelligent classification and recognition.

## 4. Conclusions

In summary, we have developed flexible strain sensors with self-assembled MXene/MWCNT/PDMS, in which MXene particles are wrapped and bridged by dense MWCNTs, forming complex sliding conductive networks and enhancing the sensors’ performance effectively. It is found that the sensor with a 2:1 MXene/MWCNT weight ratio with 12 cycle numbers achieved an impressive sensitivity (GF = 646) and high stability, even after 1800 cycles at 10% strain with no substantial increase in relative resistance change. In addition, a rapid response time of 280 ms and a detection limit of 0.05% could also be obtained. The above potential sensing performance enables the self-assembled MXene/MWCNT/PDMS strain sensor to be highly suitable for applications involving human motion and healthcare detection, including joint movements, facial expressions, and subtle pulse actions. It is also applied to wearable smart devices, in which the CNN algorithm is utilized to identify 15 gestures, and the final recognition rate is up to 95%. Our findings may hold considerable significance for the future advancement of flexible strain sensors, which could inform potential applications in areas such as human health monitoring, speech recognition, and human–machine interaction.

## Figures and Tables

**Table 1:** Comparing the performance of strain sensors with either MXene or MWCNTs as fillers.

Composition	Gauge Factor	Response Range	Sensing Repeatability	Ref.
MWCNT/PDMS	513	5%	100	[45]
MWCNT/PDMS	4.3	30%	300	[46]
Graphene/MWCNT	181.36	7.5%	5000	[47]
MWCNT/PDMS	9	40%	1000 (10%)	[48]
MWCNT/Ecoflex		1300%	6000 (100%)	[49]
CNTs/PDMS	7.22	40%	500	[50]
MXene/polyurethane	228	150%	3200 (10%)	[51]
MXene/PDMS	178	53%	5000 (20%)	[52]
MXene/PDMS	7000	5%	450 (0.5%)	[53]
MXene/MWCNT/PDMS	646	40%	1800 (10%)	This work

## Data Availability

The data that support the findings of this study are available from the corresponding author upon reasonable request.

## References

[1] Lee J.H., Cho K., Kim J.K. (2024). Age of flexible electronics: Emerging trends in soft multifunctional sensors. Adv. Mater..

[2] Tian H., Liu C., Hao H., Wang X., Chen H., Ruan Y., Huang J. (2024). Recent advances in wearable flexible electronic skin: Types, power supply methods, and development prospects. J. Biomater. Sci. Polym. Ed..

[3] Hong W., Guo X., Zhang T., Zhu X., Su Z., Meng Y., Zhao Y., Xu D., Pan J., Huang Y. (2024). Dual bionic-inspired stretchable strain sensor based on graphene/multi-walled carbon nanotubes/polymer composites for electronic skin. Compos. Part A Appl. Sci. Manuf..

[4] Zhang L., Zhou M., He Y., Wang L., Song H., Du H., Liu H., Liu C. (2024). Flexible porous non-woven silk fabric based conductive composite for efficient multimodal sensing. Chem. Eng. J..

[5] Guo X., Liu T., Tang Y., Li W., Liu L., Wang D., Zhang Y., Zhang T., Zhu X., Guan Y. (2024). Bioinspired Low Hysteresis Flexible Pressure Sensor Using Nanocomposites of Multiwalled Carbon Nanotubes, Silicone Rubber, and Carbon Nanofiber for Human–Computer Interaction. ACS Appl. Nano Mater..

[6] Zhai K., Wang H., Ding Q., Wu Z., Ding M., Tao K., Yang B.R., Xie X., Li C., Wu J. (2023). High-Performance Strain Sensors Based on Organohydrogel Microsphere Film for Wearable Human–Computer Interfacing. Adv. Sci..

[7] Wang H., Jiang Y., Ma Z., Shi Y., Zhu Y., Huang R., Feng Y., Wang Z., Hong M., Gao J. (2023). Hyperelastic, robust, fire-safe multifunctional MXene aerogels with unprecedented electromagnetic interference shielding efficiency. Adv. Funct. Mater..

[8] Wang J., Cui X., Song Y., Chen J., Zhu Y. (2023). Flexible iontronic sensors with high-precision and high-sensitivity detection for pressure and temperature. Compos. Commun..

[9] Huang C.-Y., Yang G., Huang P., Hu J.-M., Tang Z.-H., Li Y.-Q., Fu S.-Y. (2023). Flexible pressure sensor with an excellent linear response in a broad detection range for human motion monitoring. ACS Appl. Mater. Interfaces.

[10] Yuan Y., Zhou J., Lu G., Sun J., Tang L. (2021). Highly stretchable, transparent, and self-adhesive ionic conductor for high-performance flexible sensors. ACS Appl. Polym. Mater..

[11] Su Y., Ma K., Yuan F., Tang J., Liu M., Zhang X. (2022). High-performance flexible piezoresistive sensor based on Ti3C2Tx MXene with a honeycomb-like structure for human activity monitoring. Micromachines.

[12] Khalid M.A.U., Chang S.H. (2022). Flexible strain sensors for wearable applications fabricated using novel functional nanocomposites: A review. Compos. Struct..

[13] Zhang X., Liu D., Liu S., Cai Y., Shan L., Chen C., Chen H., Liu Y., Guo T., Chen H. (2024). Toward Intelligent Display with Neuromorphic Technology. Adv. Mater..

[14] Afsarimanesh N., Nag A., Sarkar S., Sabet G.S., Han T., Mukhopadhyay S.C. (2020). A review on fabrication, characterization and implementation of wearable strain sensors. Sens. Actuators A Phys..

[15] Cheng H.-W., Yan S., Shang G., Wang S., Zhong C.-J. (2021). Strain sensors fabricated by surface assembly of nanoparticles. Biosens. Bioelectron..

[16] Choi Y.K., Park T., Lee D.H.D., Ahn J., Kim Y.H., Jeon S., Han M.J., Oh S.J. (2022). Wearable anti-temperature interference strain sensor with metal nanoparticle thin film and hybrid ligand exchange. Nanoscale.

[17] Ketelsen B., Schlicke H., Schulze V.R., Bittinger S.C., Wu S.D., Hsu S.H., Vossmeyer T. (2023). Nanoparticle-Based Strain Gauges: Anisotropic Response Characteristics, Multidirectional Strain Sensing, and Novel Approaches to Healthcare Applications. Adv. Funct. Mater..

[18] Liu X., Liang X., Lin Z., Lei Z., Xiong Y., Hu Y., Zhu P., Sun R., Wong C.P. (2020). Highly sensitive and stretchable strain sensor based on a synergistic hybrid conductive network. ACS Appl. Mater. Interfaces.

[19] Zhang K., Jiang W., Li X., Gao X. (2022). Highly stretchable and sensitive strain sensors based on modified PDMS and hybrid particles of AgNWs/graphene. Nanotechnology.

[20] Zhang P., Chen Y., Li Y., Zhao Y., Wang W., Li S., Huang L. (2019). Flexible piezoresistive sensor with the microarray structure based on selfassembly of multi-walled carbon nanotubes. Sensors.

[21] He Y., Wu D., Zhou M., Zheng Y., Wang T., Lu C., Zhang L., Liu H., Liu C. (2021). Wearable strain sensors based on a porous polydimethylsiloxane hybrid with carbon nanotubes and graphene. ACS Appl. Mater. Interfaces.

[22] He Y., Lu X., Wu D., Zhou M., He G., Zhang J., Zhang L., Liu H., Liu C. (2023). CNT/PDMS conductive foambased piezoresistive sensors with low detection limits, excellent durability, and multifunctional sensing capability. Sens. Actuators A Phys..

[23] Aikawa S., Zhao Y., Yan J. (2022). Development of High-Sensitivity Electrically Conductive Composite Elements by Press Molding of Polymer and Carbon Nanofibers. Micromachines.

[24] Bi P., Zhang M., Li S., Lu H., Wang H., Liang X., Liang H., Zhang Y. (2023). Ultra-sensitive and wide applicable strain sensor enabled by carbon nanofibers with dual alignment for human machine interfaces. Nano Res..

[25] Lu X., Qin Y., Chen X., Peng C., Yang Y., Zeng Y. (2022). An ultra-wide sensing range film strain sensor based on a branch-shaped PAN-based carbon nanofiber and carbon black synergistic conductive network for human motion detection and human–machine interfaces. J. Mater. Chem. C.

[26] Zha A.-Y., Zha Q.-B., Li Z., Zhang H.-M., Ma X.-F., Xie W., Zhu M.-S. (2023). Surfactant-enhanced electrochemical detection of bisphenol A based on Au on ZnO/reduced graphene oxide sensor. Rare Met..

[27] Wang S., Chen F., Li Z., Tao H., Qu L., Li J., Zhu M., Zha Q. (2023). A graphene oxide/Zn-metal organic framework electrochemical sensor for acetaminophen detection. Surf. Interfaces.

[28] You X., Yang J., Wang M., Hu J., Ding Y., Zhang X., Dong S. (2019). Graphene-based fiber sensors with high stretchability and sensitivity by direct ink extrusion. 2D Mater..

[29] Luan H., Zhang D., Xu Z., Zhao W., Yang C., Chen X. (2022). MXene-based composite double-network multifunctional hydrogels as highly sensitive strain sensors. J. Mater. Chem. C.

[30] Chae A., Murali G., Lee S.Y., Gwak J., Kim S.J., Jeong Y.J., Kang H., Park S., Lee A.S., Koh D.Y. (2023). Highly Oxidation-Resistant and Self-Healable MXene-Based Hydrogels for Wearable Strain Sensor (Adv. Funct. Mater. 24/2023). Adv. Funct. Mater..

[31] Ni Q.-Y., He X.-F., Zhou J.-L., Yang Y.-Q., Zeng Z.-F., Mao P.-F., Luo Y.-H., Xu J.-M., Jiang B., Wu Q. (2024). Mechanical tough and stretchable quaternized cellulose nanofibrils/MXene conductive hydrogel for flexible strain sensor with multi-scale monitoring. J. Mater. Sci. Technol..

[32] Yang Y., Cao Z., He P., Shi L., Ding G., Wang R., Sun J. (2019). Ti3C2Tx MXene-graphene composite films for wearable strain sensors featured with high sensitivity and large range of linear response. Nano Energy.

[33] Yang H., Xiao X., Li Z., Li K., Cheng N., Li S., Low J.H., Jing L., Fu X., Achavananthadith S. (2020). Wireless Ti3C2T x MXene strain sensor with ultrahigh sensitivity and designated working windows for soft exoskeletons. ACS Nano.

[34] Nag A., Alahi M.E.E., Mukhopadhyay S.C., Liu Z. (2021). Multi-walled carbon nanotubes-based sensors for strain sensing applications. Sensors.

[35] Nie M., Ren X., Wen L., Han L., Wang J., Su S. (2021). Highly sensitive and large range strain sensor based on synergetic effects with double conductive layer structures. Sens. Actuators A Phys..

[36] Xing H., Li X., Lu Y., Wu Y., He Y., Chen Q., Liu Q., Han R.P. (2022). MXene/MWCNT electronic fabric with enhanced mechanical robustness on humidity sensing for real-time respiration monitoring. Sens. Actuators B Chem..

[37] Kalambate P.K., Dhanjai, Sinha A., Li Y., Shen Y., Huang Y. (2020). An electrochemical sensor for ifosfamide, acetaminophen, domperidone, and sumatriptan based on self-assembled MXene/MWCNT/chitosan nanocomposite thin film. Microchim. Acta.

[38] Chen S., Xu J., Shi M., Yu Y., Xu Q., Duan X., Gao Y., Lu L. (2021). Polydopamine bridged MXene and NH2-MWCNTs nanohybrid for high-performance electrochemical sensing of Acetaminophen. Appl. Surf. Sci..

[39] Chachuli S.A.M., Hamidon M.N., Ertugrul M., Mamat M.S., Coban O., Tuzluca F.N., Yesilbag Y.O., Shamsudin N. (2021). Effects of MWCNTs/graphene nanoflakes/MXene addition to TiO2 thick film on hydrogen gas sensing. J. Alloys Compd..

[40] Qiu A., Li P., Yang Z., Yao Y., Lee I., Ma J. (2019). A path beyond metal and silicon: Polymer/nanomaterial composites for stretchable strain sensors. Adv. Funct. Mater..

[41] Wang H., Liu J., Cui H., Liu Y., Zhu J., Wang H., Song G., Li Z., Chen D. (2021). Strain sensor with high sensitivity and large response range based on self-assembled elastic-sliding conductive networks. ACS Appl. Electron. Mater..

[42] Yang L., Li Y., Wang H., Wei S., Li Z., Liu Y., Chen D., Guo Q., Sun X. (2022). Flexible assembled tactile sensor with freely integration design. Smart Mater. Struct..

[43] Chen T., Wang J., Wu X., Li Z., Yang S. (2021). Ethanediamine induced self-assembly of long-range ordered GO/MXene composite aerogel and its piezoresistive sensing performances. Appl. Surf. Sci..

[44] Xu X., Chen Y., He P., Wang S., Ling K., Liu L., Lei P., Huang X., Zhao H., Cao J. (2021). Wearable CNT/Ti3C2T x MXene/PDMS composite strain sensor with enhanced stability for real-time human healthcare monitoring. Nano Res..

[45] Huang K., Ning H., Hu N., Liu F., Wu X., Wang S., Liu Y., Zou R., Yuan W., Wu L. (2020). Ultrasensitive MWCNT/PDMS composite strain sensor fabricated by laser ablation process. Compos. Sci. Technol..

[46] Abshirini M., Charara M., Liu Y., Saha M., Altan M.C. (2018). 3D printing of highly stretchable strain sensors based on carbon nanotube nanocomposites. Adv. Eng. Mater..

[47] Lu S., Ma J., Ma K., Wang X., Wang S., Yang X., Tang H. (2019). Highly sensitive graphene platelets and multi-walled carbon nanotube-based flexible strain sensor for monitoring human joint bending. Appl. Phys. A.

[48] Fu X., Ramos M., Al-Jumaily A.M., Meshkinzar A., Huang X. (2019). Stretchable strain sensor facilely fabricated based on multi-wall carbon nanotube composites with excellent performance. J. Mater. Sci..

[49] Zhang Y., Zhu X., Liu Y., Liu L., Xu Q., Liu H., Wang W., Chen L. (2021). Ultra-stretchable monofilament flexible sensor with low hysteresis and linearity based on MWCNTs/Ecoflex composite materials. Macromol. Mater. Eng..

[50] Li T., Li J., Zhong A., Han F., Sun R., Wong C.-P., Niu F., Zhang G., Jin Y. (2020). A flexible strain sensor based on CNTs/PDMS microspheres for human motion detection. Sens. Actuators A Phys..

[51] Yang K., Yin F., Xia D., Peng H., Yang J., Yuan W. (2019). A highly flexible and multifunctional strain sensor based on a network-structured MXene/polyurethane mat with ultra-high sensitivity and a broad sensing range. Nanoscale.

[52] Yang Y., Shi L., Cao Z., Wang R., Sun J. (2019). Strain sensors with a high sensitivity and a wide sensing range based on a Ti3C2Tx (MXene) nanoparticle–nanosheet hybrid network. Adv. Funct. Mater..

[53] Kedambaimoole V., Kumar N., Shirhatti V., Nuthalapati S., Sen P., Nayak M.M., Rajanna K., Kumar S. (2020). Laser-induced direct patterning of free-standing Ti3C2–MXene films for skin conformal tattoo sensors. ACS Sens..

[54] Chittibabu S.K., Chintagumpala K. (2023). Evolution of 2D materials conducive to the wearable physical sensors for structural health assessment. Microelectron. Eng..

